# Bacterial concentration and *Campylobacter spp*. quantification differ when fresh or ultra-frozen samples are analysed over time using molecular biology and culture-based methods

**DOI:** 10.1371/journal.pone.0274682

**Published:** 2022-09-16

**Authors:** Farina Khattak, Salvatore Galgano, Jos Houdijk

**Affiliations:** Monogastric Science Research Centre, SRUC, Edinburgh, United Kingdom; Georgia Southern University, UNITED STATES

## Abstract

The study aimed to delineate the robustness of the culture-based and molecular biology methods to assess the total bacterial concentration and *Campylobacter jejuni (C*. *jejuni)* quantification in caecal content, analysed as fresh or after being stored immediately at ultra-low (-80°C) temperature at different time points (for 3, 7, 14, 28 and 62 days post collection). The caecal content was collected from birds that were artificially colonised with *C*. *jejuni (in-vivo)*, *and quantification was performed using both colony-forming unit (CFU)* and qPCR. The results showed that storage time affected the output of culture-based analyses but mostly did not alter concentration retrieved via qPCR. After an initial ~4.5 log_10_ reduction in CFU observed from fresh (day 0) to frozen samples, bacterial concentration retrieved with culture-based methods seemed to be constant in samples frozen for 3 to 62 days, indicating a possible threshold for *C*. *jejuni* loss of viability due to effect of storage temperature. Ranking order analyses, revealed that the molecular biology technique was able to attribute somewhat the same relative *C*. *jejuni* concentrations to the samples analysed via qPCR. However, day 0 measurements from culture-based methods were associated with the absence of or negatively weak correlations with the rest of the time points, but ranking order was maintained from day 3 onwards. On the other hand, ranking order correlations were less constant when measuring total bacterial concentration through qPCR. The study suggests that if biological samples can’t be analysed as fresh (immediately after collection) and have to be stored prior to analysis, then storage at -80°C samples be recommended to avoid the temporal-dependent effects on *C*. *jejuni* concentrations. In addition, irrespective of the method of analysis, an initial loss of CFU must be factored in when interpreting the results obtained from frozen samples.

## Introduction

According to the European Union One Health 2019 Zoonoses report, campylobacteriosis remains the first most reported bacterial zoonoses in humans [1]. *C*. *jejuni*, most commonly regarded as the human foodborne pathogen involved, is an obligate microaerophilic and grows optimally at ~42°C, although minimal growth temperature varies in the range of 31 to 36°C, whilst growth ceases abruptly around 30°C [2–4]. *Campylobacter* spp. are fastidious organisms that require low partial oxygen tension [5], are preferentially extracellular, albeit intracellular viability has been shown [6] and are highly susceptible to a number of environmental conditions [7]. Despite their fastidious growth requirements, *C*. *jejuni* manages to survive under conditions nonpermissive to growth, which is highly relevant to food safety and public health. *C*. *jejuni* can survive on chicken skin and remain in viable but non-culturable stage for up to four months at 4°C [8, 9] and continue to survive for up to 7 months based on signs of cellular integrity, respiratory activity and intact DNA content [10]. Studies show that storage conditions and duration jeopardise *Campylobacter* spp. enumeration by culture-based methods [11] and thus could result in false-negative or positives.

Though widely used, traditional culture-based detection methods require 18 to 96 hours and sometimes are prone to false-negative, mostly due to *Campylobacter* spp. sensitivity to different culture conditions and by stress-driven "viable but non-culturable" status in which the bacterium may be found [12]. Due to such reasons, the use of molecular methods, and especially quantitative polymerase chain reaction (qPCR), represents a valid alternative for *Campylobacter* detection [13]. Such protocols eliminate the incubation step typical of culture-based methods by targeting genetic material, hereby reducing measurement time to as little as 2 hours, allowing quick and unambiguous detection and identification of thermophilic *Campylobacter* [14].

Campylobacteriosis is frequently associated with the handling and consumption of poultry meat [3]. To reduce campylobacteriosis, monitoring the levels of *Campylobacter* spp. in fresh and frozen chicken at the retail sale is a key priority. In most cases for enumeration of *Campylobacter*, chicken samples are transported to distant laboratories in a frozen state as testing fresh samples is not possible due to the time it takes to transport samples to the lab. Though culture base detection methods are recognised as the gold standard, the question remains if the state in which samples are kept before analysis has an impact on *Campylobacter* quantification. Caecal samples from birds exposed to commercially relevant levels of *C*. *jejuni* were used in this study with the aim of assessing the effect of sample state (Fresh or -80°C) and longevity (up to 62 days in storage) impact quantification of *Campylobacter* and bacterial concentration, using both culture-based methods and molecular biology methods.

## Materials and methods

The caecal content was collected from birds that were artificially infected with *C*. *jejuni*, and quantification was performed on both fresh samples and their frozen aliquots that were stored immediately at -80°C for 3, 7, 14, 28 and 62 days (storage time points) post collection and through both colony-forming unit (CFU) enumeration and qPCR.

### Animal experiment and sample preparation

In observance of the ethical principle of reduction, a total of 24 thirty-five-day-old male Ross 308 broilers (i.e., 4 birds from 6 pens) were humanely culled during a parallel study [15] and were used to undertake the analysis presented here. On day 20 of the animal trial, chickens were artificially infected with *C*. *jejuni* ATCC33291 strain (7 x 10^6^ CFU/ml) through seeded litter tray procedure [16]. Fifteen days thereafter, birds were humanly culled via cervical dislocation, and caecal content from the four birds per pen was pooled, forming six pooled samples. These were then separated into 12 different aliquots per pen (i.e., 72 aliquots in total, Table 1), and two aliquots per pen were immediately used for CFU enumeration and DNA isolation (i.e., experimental day 0), respectively. The remaining aliquots (i.e., 10 per pen) were immediately stored at -80°C, and at time points day 3, 7, 14, 28 and 62, two aliquots per pen were thawed on ice for ~30 minutes and processed to carry out CFU enumeration and DNA isolation. Time points were chosen to represent approximately doubling storage time over time. CFU enumeration was carried out after 48 hours of incubation at 41°C ±1.5°C from each time point, whereas isolated gDNA was stored at -80°C after each time-point extraction until qPCR was performed for all the samples contemporarily. To avoid cofounding factors, temperature in the lab was electronically controlled and equipment’s such as pipettes were pretested prior to analysis. The number of replicates used in this study was within the range of values known to give an appropriate probability (power) of the objectives of the experiment being met according to similar study design conducted previously.

**Table 1:** Aliquots per pen and experimental schedule.

		Aliquot number											
		1	2	3	4	5	6	7	8	9	10	11	12
		Day 0	Day 3	Day 7	Day 14	Day 28	Day 62	Day 0	Day 3	Day 7	Day 14	Day 28	Day 62
**Pen number**	**1**	CFU enumeration (culture-based analysis)						qPCR (molecular biology-based analysis)					
	**2**												
	**3**												
	**4**												
	**5**												
	**6**												

### Blinding

Animal trial facility staff and laboratory technicians in charge of animal care, sample collection and analytical analysis were blinded to treatment allocations. Blinding of treatments to study personnel was done by randomly assigning a unique number to each treatment. These unique numbers were used on the feed bags, pen labelling (animal study) and on sample aliquots. Test facility staff involved in the mixing and blinding of the feeds and samples did not perform any study observations.

### CFU enumeration

One gram of pooled caecal content was suspended in 9 ml of sterile phosphate buffered saline (PBS) and thoroughly mixed, thus further ten-fold serially diluted in sterile PBS. Selected dilutions were plated (100μl) onto Charcoal Cefoperozone Deoxycholate agar (CCDA) (Oxoid) and incubated at 41°C ±1.5 for 48 hours in hermetic jars containing microaerophilic generation bags (CampyGen, Oxoid). For fresh samples, plating was carried out soon after collection and CFU enumeration was performed after 48 hours. Whereas frozen samples were thawed on ice for ~30 minutes before carrying out the serial dilutions and therefore plating, with CFU enumeration being performed 48 hours after that. CFU per ml was calculated by multiplying the dilution factor for the ratio between the number of colonies observed on the plates and the volume plated. To facilitate comparison through all the analyses, *C*. *jejuni* concentration output of the CFU enumeration was expressed in bacteria/g.

### Total DNA isolation

A total of ~0.25g of pooled caecal samples were transferred in PowerBead tubes of the DNeasy PowerSoil Kit (QIAGEN, Part no. 12888–100). After adding 60μl of solution C1 from the same kit, tubes were placed in a FastPrep-24^TM^ 5G homogeniser (MP Biomedicals) for 55 seconds at 5.5 m/s. QIAGEN 12888–100 manufacturer instructions were followed to isolate total DNA, which was immediately stored at -80°C until further analysis.

### Absolute qPCR quantification

Absolute quantification linear regression model was based on a linear plasmid-standard curve built through nine serial 10-fold dilutions, allowing quantification of the total number of bacteria, *Campylobacter* spp. and *C*. *jejuni*, as depicted in Table 2. Plasmid DNA was linearised to reduce overestimation biases due to supercoiled plasmid standard [17], and three different plasmids were used to quantify different targets, respectively.

**Table 2:** List of primers used in this study.

Primer	Target (target gene)	Sequence (5’→3’)	Annealing temp. (°C)	Amplicon length (bp)	Reference
*C_sppF*	*Campylobacter* spp. (16S rRNA gene)	CACGTGCTACAATGGCATATACAA	60	77	[18]
*C_sppR*		CCGAACTGGGACATATTTTATAGATTT			
*C_jejF*	*Campylobacter jejuni* (VS1)	GAATGAAATTTTAGAATGGGG	60	358	[13]
*C_jejR*		GATATGTATGATTTTATCCTGC			
341F	Total bacteria (16S rRNA gene, V3 region)	CCTACGGGAGGCAGCAG	60	194	[19]
518R		ATTACCGCGGCTGCTGG			

#### Standard curve preparation

A total of 1μl of caecal content genomic DNA was used as a template in three different PCR reactions using primers depicted in Table 2. Reaction components included KAPA Taq ReadyMix with dye (1X, Kapa Biosystems), F and R primers (0.2μM)and nuclease-free water up to a volume of 25μl. Cycling conditions were; 95°C initial denaturation (3 minutes), 35 cycles of denaturation at 95°C for 30 seconds, annealing at 60°C for 30 seconds and elongation at 72°C for 1 minute followed by a final elongation cycle at 72°C for 10 minutes.

Amplicons were excised from 1.5% agarose gel after electrophoresis at 100V for 80 minutes, purified using Wizard^®^ SV Gel and PCR Clean-Up System (Promega), cloned into pCR2.1 plasmid vector (1:1 insert to vector ratio; TA Cloning Kit, Thermo Fisher Scientific). and transformed into chemically competent One shot^®^ INVαF’ *E*. *coli* cells (Thermo Fisher Scientific) by heat shock. Plasmids were isolated from positive X-gal colonies after overnight incubation at 37°C (QIAprep Miniprep kit), and insert presence was verified by both EcoRI (New England BioLabs) digestion and Sanger sequencing (DNA Sequencing and Services, Medical Sciences Institute, School of Life Sciences, University of Dundee). Thus, linearisation was carried out using 5 units of HindIII (New England BioLabs) and 1X of CutSmart^®^ buffer (New England BioLabs) in 50μl total volume, followed by purification after electrophoretic separation using Wizard^®^ SV Gel and PCR Clean-Up System (Promega).

#### qPCR reaction conditions

All reactions were carried out in 20μl containing 1X Brilliant III Ultra-Fast SYBR Green qPCR Master Mix (Agilent technologies), 100nM of each primer (Table 2), 1ng of DNA template and nuclease-free water (QIAGEN, Hilden, Germany). Cycling conditions (Mx3000thermocycler, Agilent Technologies) were 95°C for 3 minutes followed by 40 cycles at 95°C for 10 seconds and annealing and fluorescence detection at 60°C for 20 seconds. Qualitative template control was performed through melting curve analysis, and all the reactions were run in triplicate, including a non-template control. Reaction efficiency based on standard curve analysis was evaluated through linear regression (r^2^), slope and efficiency, whose values throughout the analyses performed were ~0.99, ~3.55 and ~95%, respectively, thus indicative of reliable technical performance.

#### qPCR data analysis

Raw gene copy number (CN) per reaction output of the qPCR linear regression model was calculated using Stratagene Mx3000P software (Agilent technologies).

Therefore, CN was converted into bacterial cells (BN) per gram of sample (BN/g) considering an average of 5.2 16S gene copies per bacterial cell, at the time of writing [20], and an average of 2.6 16S copies per *Campylobacter spp*. cell, at the time of writing [20], and *C*. *jejuni* CN to BN conversion was based on the assumption of one *VS1* gene copy per cell. Eq (1) below was thus used to calculate BN/g [21].

$$\frac{BN*C*DV}{S*V}.$$ (1)

Where C and DV were concentration and dilution volume of extracted DNA, respectively, whilst S was the amount (ng) of DNA subjected to qPCR and V was the volume (ng) of sample used to isolate DNA [21].

#### Ranking order analysis

The ranking order analysis was carried out by Spearman’s rank-order correlation and this allowed to assess whether the different techniques used (e.g CFU enumeration and qPCR quantification) detected the same realtaive contarations through out the same time points.

### Statistical analysis

Analyses were carried out in R (version 4.0.3 [22]), using RStudio (version 1.4.1103). Both CFU and qPCR data were subjected to factorial ANOVA [22] to assess i) whether storage time and ii) different techniques influenced the Log_10_ transformed concentration of *Campylobacter* spp., *C*. *jejuni* and total bacteria (the targets). Aliquot was the experimental unit for all analysis. No outliers were removed prior to statistical analysis. The distribution was all normal as checked via OO-plot analysis. Post hoc analysis was carried out through Tukey Honest Significant Differences (HSD) in R [22].

For ranking order analysis, spearman’s rank-order correlation (*ρ*) was carried out in R [22]. The variables ananalysed were the ranking order of the bacterial concentration throughout measurements at different time points and from different methods of analyses. Due to sample size <10, specific probability tables were used for hypothesis testing [23], and ρ threshold of 0.886 was considered corresponding to a significance level (α) of 0.05 for a two-tailed test [24].

### Ethical approval

This research used caecal samples of birds derived from an *in vivo* study (POU AE-13-2020), which was carried out under the Animal Scientific Act (1986). All the procedures used in this experiment were approved by the ethical review committee of Scotland’s Rural College (SRUC).

## Results

Results of the factorial ANOVA analysis showed that normalised log_10_ BN was significantly associated (P<0.0005) to both factors (i.e., storage time and analytical technique). Moreover, a significant interaction, F (15,100) = 5.815, P<0.0001, was found between storage time and analytical technique as described below.

### Effect of storage time on bacterial quantification

**Fig 1:**
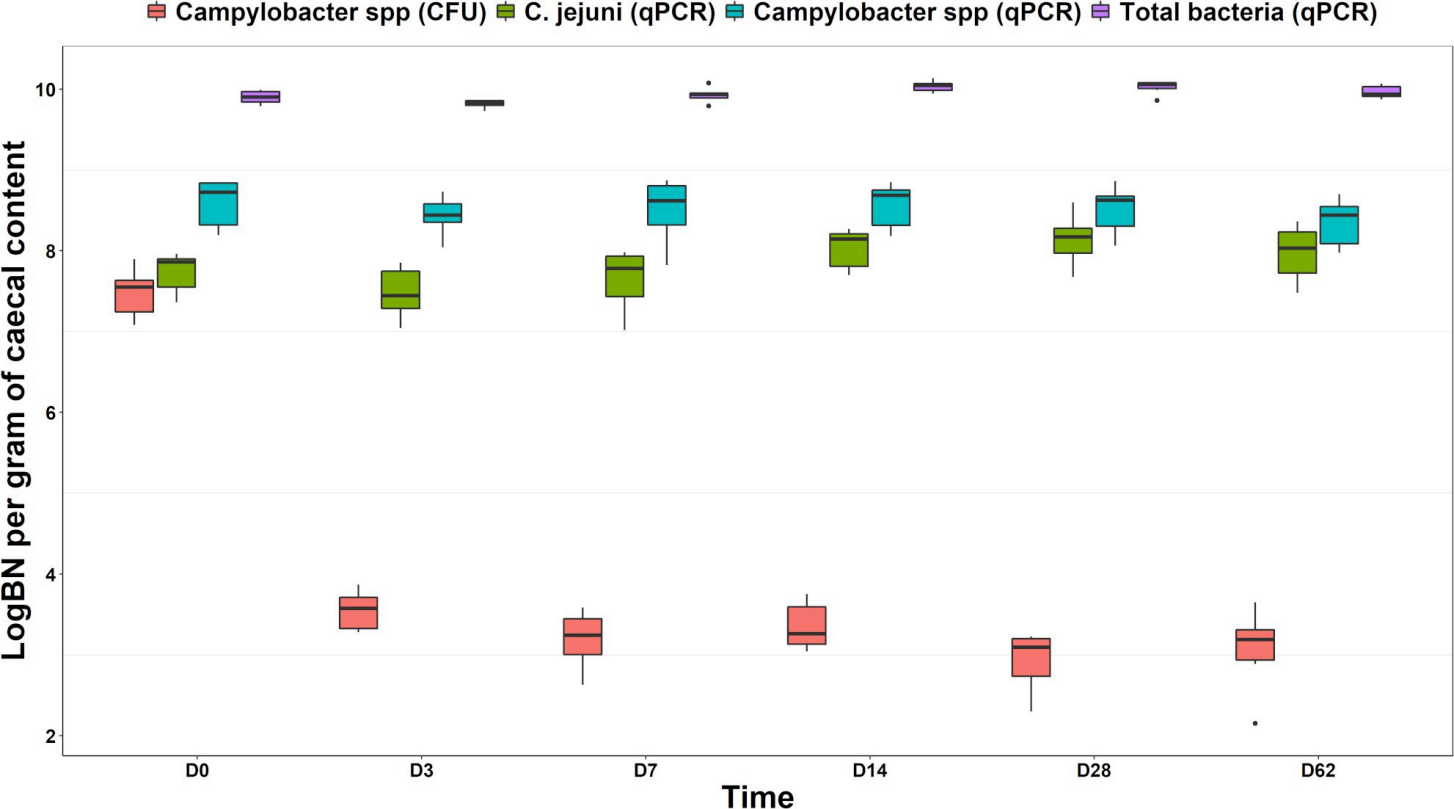
Log_10_ bacterial concentration of *Campylobacter* genus, *C*. *jejuni* and total bacteria measured via qPCR and culture-based test throughout the six experimental time points.

*Campylobacter* spp. concentration measured through CFU enumeration at time 0 (3.68∙10^7^ bacteria/g) was ~4.5 Log_10_ higher according to Tukey HSD posthoc analysis than the measurements at the rest of the time points (p < 0.05), (Table 3 and Fig 1). However, after an initial reduction to 4.04∙10^3^ bacteria/g at day 3, *Campylobacter* spp concentration calculated through culture method analysis remained constant (p >0.05) to an average of 1.89∙10^3^ bacteria/g from day 7 to day 62.

**Table 3:** Time-point Log_10_ bacteria/g differences amongst different analyses (i.e., columns -rows and letters from A to D). Statistically significant differences are indicated in bold and with the symbol "*".

**A**						***B***					
***Campylobacter* spp. (CFU, n = 36)**						***C*. *jejuni* (qPCR, n = 36)**					
	**D0**	**D3**	**D7**	**D14**	**D28**		**D0**	**D3**	**D7**	**D14**	**D28**
**D3**	**-3.93** ^*^					**D3**	-0.26				
**D7**	**-4.29** ^*^	-0.36				**D7**	-0.09	0.17			
**D14**	**-4.13** ^*^	-0.20	0.16			**D14**	0.30	0.55	0.39		
**D28**	**-4.55** ^*^	-0.62	-0.26	-0.42		**D28**	0.40	**0.66** ^*^	0.50	0.11	
**D62**	**-4.42** ^*^	-0.49	-0.13	-0.29	0.14	**D62**	0.24	0.49	0.33	-0.06	-0.17
**C**						**D**					
***Campylobacter* spp. (qPCR, n = 36)**						**Total bacteria (qPCR, n = 36)**					
	**D0**	**D3**	**D7**	**D14**	**D28**		**D0**	**D3**	**D7**	**D14**	**D28**
**D3**	-0.16					**D3**	-0.08				
**D7**	-0.09	0.07				**D7**	0.03	0.11			
**D14**	-0.03	0.13	0.06			**D14**	0.14	0.22	0.11		
**D28**	-0.08	0.08	0.01	-0.05		**D28**	0.12	0.21	0.10	-0.01	
**D62**	-0.24	-0.08	-0.15	-0.21	-0.16	**D62**	0.06	0.14	0.03	-0.07	-0.06

On the other hand, *Campylobacter* spp. concentration measured via qPCR remained stable (p >0.05) from day 0 to day 62 with an average of ~3.8∙10^8^ bacteria/g throughout. The concentration of *C*. *jejuni* (qPCR) was also statistically unchanged (p >0.05) throughout the majority of the aliquots analysed, although the differences between some of the time points were somewhat more noticeable, with day 3 concentration (3.71∙10^7^ bacteria/g) being ~0.5 Log_10_ lower than measurements at days 14 and 62 (~1.21∙10^8^ bacteria/g) and significantly different (p <0.05) than concentration retrieved at day 28 (1.7∙10^8^ bacteria/g, Table 3 and Fig 1).

### Effect of analysis method on bacterial quantification

Tukey HSD posthoc analysis of the factorial ANOVA revealed that *C*. *jejuni* concentration on fresh samples (i.e., day 0) measured through qPCR (6.14∙10^7^ bacteria/g) was rather similar (p = 0.998) to *Campylobacter* spp. concentration measured through culture-based analysis (3.68∙10^7^ bacteria/g). However, the two analyses retrieved different figures from day 3 through to day 62 (p = 2.41∙10^−14^) due to the reduced levels described above for the CFU enumeration. At day 0 though, *Campylobacter* spp. concentration measured via qPCR (4.68∙10^8^ bacteria/g) was higher than both *Campylobacter* spp. (CFU enumeration, 3.68∙10^7^ bacteria/g, p = 2.91∙10^−7^) and *C*. *jejuni* (qPCR, 6.14∙10^7^ bacteria/g, p = 3.14∙10^−4^). Indeed, qPCR revealed a higher concentration of *Campylobacter* genus at all time points when compared to culture-based analysis (p = 4.85∙10^−8^), whereas it registered >0.85 Log_10_ bacteria/g higher levels than qPCR for *C*. *jejuni* during the first three-time points (p = 2.10∙10^−4^). However, qPCR concentrations of *Campylobacter* genus and *C*. *jejuni* were comparable (p = 0.22) at day 14 (difference: 0.53 Log_10_ bacteria/g), day 28 (difference: 0.37 Log_10_ bacteria/g, p = 0.86) and day 62 (difference: 0.38 Log_10_ bacteria/g, p = 0.83). (Table 4 and Fig 1). Expectedly, total bacterial concentration (qPCR) was higher (p <0.05) than *Campylobacter spp* (qPCR and CFU) and *C*. *jejuni* (qPCR) through all the analysed time points.

**Table 4:** Log_10_ bacteria concentration amongst different analyses throughout the six experimental time points (i.e., columns -rows and letters from A to F) Statistically, significant differences (Tukey HSD) are indicated in bold and with the symbol *.

**A**				**B**			
**D0 (n = 24)**				**D3 (n = 24)**			
	${BN}_{CFU}^{C.spp}$	${BN}_{qPCR}^{C.jejuni}$	${BN}_{qPCR}^{C.spp.}$		${BN}_{CFU}^{C.spp}$	${BN}_{qPCR}^{C.jejuni}$	${BN}_{qPCR}^{C.spp.}$
${BN}_{qPCR}^{C.jejuni}$	0.25			${BN}_{qPCR}^{C.jejuni}$	3.93*		
${BN}_{qPCR}^{C.spp.}$	1.11*	0.86*		${BN}_{qPCR}^{C.spp.}$	4.88*	0.96*	
${BN}_{qPCR}^{16S}$	2.42*	2.16*	1.31*	${BN}_{qPCR}^{16S}$	6.27*	2.34*	1.38*
**C**				**D**			
**D7 (n = 24)**				**D14 (n = 24)**			
	${BN}_{CFU}^{C.spp}$	${BN}_{qPCR}^{C.jejuni}$	${BN}_{qPCR}^{C.spp.}$		${BN}_{CFU}^{C.spp}$	${BN}_{qPCR}^{C.jejuni}$	${BN}_{qPCR}^{C.spp.}$
${BN}_{qPCR}^{C.jejuni}$	4.45*			${BN}_{qPCR}^{C.jejuni}$	4.68*		
${BN}_{qPCR}^{C.spp.}$	5.31*	0.86*		${BN}_{qPCR}^{C.spp.}$	5.21*	0.53	
${BN}_{qPCR}^{16S}$	6.74*	2.28*	1.42*	${BN}_{qPCR}^{16S}$	6.68*	2.00*	1.47*
**E**				**F**			
**D28 (n = 24)**				**D62 (n = 24)**			
	${BN}_{CFU}^{C.spp}$	${BN}_{qPCR}^{C.jejuni}$	${BN}_{qPCR}^{C.spp.}$		${BN}_{CFU}^{C.spp}$	${BN}_{qPCR}^{C.jejuni}$	${BN}_{qPCR}^{C.spp.}$
${BN}_{qPCR}^{C.jejuni}$	5.21*			${BN}_{qPCR}^{C.jejuni}$	4.91*		
${BN}_{qPCR}^{C.spp.}$	5.58*	0.37		${BN}_{qPCR}^{C.spp.}$	5.29*	0.38	
${BN}_{qPCR}^{16S}$	7.10*	1.89*	1.51*	${BN}_{qPCR}^{16S}$	6.90*	1.99*	1.61*

### Ranking order analysis

#### Effect of storage time on ranking-order

The analysis reveals whether the same technique was able to detect the same ranking order through different time points, thus depicting eventual similarities between two ranking orders (i.e., ρ = 1), absence of correlations (i.e., ρ = 0) or opposite ranking (i.e., ρ = -1). Statistically significant correlations were calculated using specific probability tables [23]. The ranking order for measurements of *Campylobacter* spp. (CFU enumeration) at day 0 was weakly-to-moderately negatively correlated to the order of the measurements compared to the rest of the time points. However, the ranking order for CFU enumeration was almost identical from day 3 to day 62, indicating a relatively constant ranking upon reduction of *Campylobacter* detected by the technique. (Table 5). On the other hand, qPCR for *Campylobacter spp*. and *C*. *jejuni* at day 0 was associated with strong to positive monotonic correlations through the rest of the time points, indicating that the technique was able to establish the same relative concentrations from day 0 onwards.

**Table 5:** Spearman’s rank-order correlation coefficient (ρ) calculated at different time points for each of the analytical methods and targets used in this study (i.e., columns -rows and letters from A to D).

**A**						***B***					
**CFU enumeration ρ matrix (n = 36)**						***C*. *jejuni* (qPCR) ρ matrix (n = 36)**					
	**D0**	**D3**	**D7**	**D14**	**D28**		**D0**	**D3**	**D7**	**D14**	**D28**
**D3**	-0.46	.	.	.	.	**D3**	0.77	.	.	.	.
**D7**	-0.14	0.75	.	.	.	**D7**	0.54	0.43	.	.	.
**D14**	-0.66	0.81	0.71	.	.	**D14**	0.49	0.26	0.94*	.	.
**D28**	-0.43	0.99*	0.71	0.83	.	**D28**	0.77	0.89	0.77	0.60	.
**D62**	-0.14	0.75	1.00*	0.71	0.71	**D62**	0.66	0.26	0.66	0.83	0.43
**C**						**D**					
***C*. *spp*. (qPCR) ρ matrix (n = 36)**						**Total bacteria (qPCR) ρ matrix (n = 36)**					
	**D0**	**D3**	**D7**	**D14**	**D28**		**D0**	**D3**	**D7**	**D14**	**D28**
**D3**	0.43	.	.	.	.	**D3**	-0.54	.	.	.	.
**D7**	0.77	0.77	.	.	.	**D7**	0.37	0.20	.	.	.
**D14**	0.83	0.71	0.94*	.	.	**D14**	0.14	0.60	0.54	.	.
**D28**	0.71	0.60	0.71	0.66	.	**D28**	-0.09	0.77	0.77	0.77	.
**D62**	0.94*	0.49	0.83	0.77	0.77	**D62**	0.43	-0.49	0.26	-0.09	-0.20

Where ρ = 1 depicts similarities between two ranking orders, ρ = 0 reflects an absence of correlations, and ρ = -1 shows opposite ranking.

A different scenario emerged from ranking order correlation analysis of total bacterial concentration (qPCR) through the six-time points. Indeed, ~50% of the correlations between ranking at different time-points were very weak to moderate, with five of them being negative correlations. In particular, only day 0 and day 62 were moderately correlated (ρ = 0.43), whilst day 0 relative concentrations were either weakly or negatively correlated to the remaining time points, indicating that qPCR for total bacteria revealed different concentration rankings from fresh to frozen samples, apart from day 62. On the other hand, the ranking order between concentrations measured between day 3, day 7 and day 28 were moderate to strongly positive (Table 5). Statistically significant correlations were calculated using specific probability tables [23] and are indicated in bold and with the (*) symbol in Table 5.

#### Effect of analysis method on ranking-order

Ranking analysis via Spearman’s coefficient was also carried out for different analysis methods at the same time point to assess whether ranking order of relative concentrations was maintained when the same or different targets were measured with different techniques for each of the time points. As shown in Table 6, the ranking order correlation was time-dependent. CFU enumeration was moderately correlated (ρ = 0.43) to qPCR for *C*. *jejuni* at day 0 and day 14, weakly correlated (0.31≥ρ≥0.26) to day 7 and day 62 and negatively correlated at day 3 and 28, albeit none of these correlations was statistically significant. Significant positive correlations were found between the ranking order of *Campylobacter* spp. (qPCR) and *C*. *jejuni* (qPCR) at day7 (ρ = 0.89), day14 (ρ = 0.89) and day62 (ρ = 0.94) and between ranking order of total bacteria (qPCR) and *C*. *jejuni* (qPCR) at day7 (ρ = 0.89). Although not always statistically significant when calculating the *p-value* specific probability tables [23], a ranking order of total bacteria (qPCR) was in all cases positively correlated with the ranking of the rest of the qPCR analyses (0.37≤ρ≤0.94).

**Table 6:** Spearman’s rank-order correlation coefficient (ρ) calculated for each of the analytical methods and targets used in this study at different time points (i.e., columns -rows and letters from A to F).

**A**				**B**			
**D0 ρ matrix (n = 24)**				**D3 ρ matrix (n = 24)**			
	${BN}_{CFU}^{C.spp.}$	${BN}_{qPCR}^{C.jejuni}$	${BN}_{qPCR}^{C.spp.}$		${BN}_{CFU}^{C.jejuni}$	${BN}_{qPCR}^{C.jejuni}$	${BN}_{qPCR}^{C.spp.}$
${BN}_{qPCR}^{C.jejuni}$	0.43	.	.	${BN}_{qPCR}^{C.jejuni}$	-0.78	.	.
${BN}_{qPCR}^{C.spp.}$	0.09	0.54	.	${BN}_{qPCR}^{C.spp.}$	-0.46	0.83	.
${BN}_{qPCR}^{16S}$	-0.54	0.03	-0.09	${BN}_{qPCR}^{16S}$	-0.06	0.43	0.77
**C**				**D**			
**D7 ρ matrix (n = 24)**				**D14 ρ matrix (n = 24)**			
	${BN}_{CFU}^{C.spp.}$	${BN}_{qPCR}^{C.jejuni}$	${BN}_{qPCR}^{C.spp.}$		${BN}_{CFU}^{C.spp.}$	${BN}_{qPCR}^{C.jejuni}$	${BN}_{qPCR}^{C.spp.}$
${BN}_{qPCR}^{C.jejuni}$	0.31	.	.	${BN}_{qPCR}^{C.jejuni}$	0.43	.	.
${BN}_{qPCR}^{C.spp.}$	0.37	0.89*	.	${BN}_{qPCR}^{C.spp.}$	0.54	0.89*	.
${BN}_{qPCR}^{16S}$	0.37	0.89*	0.83	${BN}_{qPCR}^{16S}$	0.71	0.43	0.37
**E**				**F**			
**D28 ρ matrix (n = 24)**				**D62 ρ matrix (n = 24)**			
	${BN}_{CFU}^{C.spp.}$	${BN}_{qPCR}^{C.jejuni}$	${BN}_{qPCR}^{C.spp.}$		${BN}_{CFU}^{C.spp.}$	${BN}_{qPCR}^{C.jejuni}$	${BN}_{qPCR}^{C.spp.}$
${BN}_{qPCR}^{C.jejuni}$	-0.49	.	.	${BN}_{qPCR}^{C.jejuni}$	0.26	.	.
${BN}_{qPCR}^{C.spp.}$	-0.37	0.83	.	${BN}_{qPCR}^{C.spp.}$	0.37	0.94*	.
${BN}_{qPCR}^{16S}$	0.09	0.54	0.54	${BN}_{qPCR}^{16S}$	0.09	0.49	0.43

Where ρ = 1 depicts similarities between two ranking orders, ρ = 0 reflects an absence of correlations, and ρ = -1 shows opposite ranking. Statistically significant (ρ ≤ 1) is indicated in bold and with the (*) symbol.

Due to I) the observed drop in *Campylobacter* spp. concentration retrieved via CFU enumeration, II) the similarities between CFU figures and *C*. *jejuni* concentration (qPCR), and III) the lack of ρ correlation between D0 and the rest of the time points within CFU-retrieved concentrations, D0 CFU ranking was compared to D3-D62 ranking calculated for *C*. *jejuni* (qPCR). It was found that ${D0}_{CFU}^{C.spp}$ was weakly correlated to ${D3}_{qPCR}^{C.jejuni}$ and ${D62}_{qPCR}^{C.jejuni}$ (average ρ~0.23) or negatively weakly correlated to the rest of the time-point rankings (average ρ~0.22), as shown in Table 7.

**Table 7:** Spearman’s rank-order correlation coefficient (ρ) calculated comparing ranking of population distribution at D0 for C. spp (CFU) and D3 to D62 for *C*. *jejuni* (qPCR).

	D0 (CFU) Vs D3 to D62 (C. jejuni, qPCR) ρ matrix (n = 36)				
	${D3}_{qPCR}^{C.jejuni}$	${D7}_{qPCR}^{C.jejuni}$	${D14}_{qPCR}^{C.jejuni}$	${D28}_{qPCR}^{C.jejuni}$	${D62}_{qPCR}^{C.jejuni}$
${D0}_{CFU}^{C.spp}$	0.2	-0.37	-0.26	-0.029	0.26

## Discussion

*Campylobacter* spp. detection and quantification in poultry samples currently rely on culture-based methods, molecular biology, or immunoassays [25]. Nevertheless, the bacterial concentration output of different techniques could be affected by factors such as the method of analysis or eventual storage time and conditions prior to analysis. Although the enumeration method of *Campylobacter* spp. is standardised [26–28], the state in which samples are kept (fresh or frozen) prior to analysis varies between studies and is sometimes not even reported. As bacterial populations, degradation of nucleic acids, proteins and other biological molecules are all affected by the storage temperature [29, 31, 32] we, therefore, hypothesise that this may affect the bacterial/campylobacter quantification and thus can give us counterfeited results.

The current study was thus designed to investigate the effect of storage conditions and storage time on *C*. *jejuni*, thus analysed as fresh and stored at -80°C for up to 62 days. Moreover, we also compared the analytical results of both culture-based and molecular biology-analyses, which are differently affected by the viability status of the bacterium and whose comparison in such experimental conditions, to the best of our knowledge, has not been explored by other studies yet. We found that CFU enumeration and qPCR retrieved the same concentration of *C*. *jejuni* on fresh samples. It must be noted that whilst CCDA is not a selective medium for *C*. *jejuni*, the number of *Campylobacter* species that can grow on this medium is limited [29, 30]. Our results showed that comparing qPCR outputs, the concentration of *Campylobacter* at the genus level was higher, as expected, than what was observed for *C*. *jejuni* only (i.e., ~92% of the total genus as an average over all the time points).

The similarity of the concentration calculated for *C*. *jejuni* (qPCR) and *Campylobacter* (CFU enumeration) at day 0 (i.e., on fresh samples), both less represented than *Campylobacter* concentration at day 0 calculated via qPCR, could reflect the absence in the samples analysed of species such as *C*. *coli*, otherwise also prone to thrive on CCDA [29]. However, we could not exclude the presence of some other species, such as *C*. *upsaliensis*, whose growth on CCDA has been shown to be involuted [31] but could still be present in the samples, as suggested by the higher concentration of the genus (qPCR) compared to *C*. *jejuni* (qPCR).

Culture-based analysis of frozen samples detected a constant lower concentration of *Campylobacter* spp., as opposed to a qPCR constant number of bacteria throughout the time points analysed, possibly indicating a decrease of viability from day 0 and day 3, which remained then constant up until day 62. Such results were not unexpected as other authors showed a similar rate of viability decline on samples frozen at -20°C for up to 14 days [10], whilst qPCR detection of the 16S rRNA gene does not discriminate for viable cells [32]. Nevertheless, a constant viable *Campylobacter* spp. concentration in frozen caecal content could raise several questions on the cause of such limited viability loss. It has been reported that the number of viable bacteria tends to decline with prolonged frozen storage (particularly if frozen storage is around -18°C, although there is usually some stabilisation after a few months where further reduction is minimal [33]. However, the bacterial diversity found in the frozen product is dependent on the initial bacterial population [33] or due to possible downstream contaminations. Usually, bacteria are more susceptible to the process of freezing, whose crystal-formation dynamics are detrimental to their cell structure [34], whereas the storage time thereafter should not theoretically impact bacterial concentration, as those cells injured at a sublethal level could recover upon thawing [33, 35].

It has also been shown that some genes could be linked to viability traits [36], therefore, it is not impossible to assume that some of the species detected after day 3 could present genomic advantages conferring after-thawing bacterial survival capabilities. Increased gene expression of chaperons such as *dnaK*, *groES*, *groEL*, and *clpB* of *Campylobacter* has been linked to its heat stress response [10] and, therefore, its ability to cause disease from retail raw chicken could be related to genetic advantages and oxidative stress resistance [37]. Together with the selective decrease in level of *Campylobacter* observed during our study at day 3, these findings could lead to further future studies targeting differences in viability genes during similar experimental conditions, similar to what was one in the past by other authors during higher temperatures [38]. Another aspect that ought to be mentioned is related to the limit of detection of both analytical techniques. Indeed the lower CFU limit of quantification is 30 CFU per plate [39], whilst the limit of detection for the qPCR reactions (i.e., for all the targets) carried out was the same as the lowest extreme standard curve point, such as one copy number per reaction, which could be converted to 0.19 bacteria if considering 5.2 average 16S gene copies per bacterial cell [20]. Samples with low bacterial concentration, such as the frozen samples examined through our study, could possibly fall under the limit of quantification for CFU enumeration, pointing towards molecular techniques as the more reliable ones in these specific cases.

Free water in meat samples is known to be converted to ice during freezing. The temperature at which water starts to freeze depends upon the concentration of solutes in the water, such as protein and carbohydrates associated with food [33]. In general, gram-negative bacteria are more susceptible to freezing injury than gram-positive organisms [33]. *Campylobacter* is especially sensitive to freezing, though there appears to be some variation in freezing tolerance between strains of *C*. *jejuni* [40, 41]. However, it is known that while freezing and frozen storage have some impact on bacteria, prolonged freezing does not make the food sterile [42]. During freezing, most microorganisms move into the unfrozen fraction of water in the food [33]. As extracellular ice forms in this fraction, the solutes become more concentrated in the unfrozen water, which causes increased water loss from the bacterial cells and exposes them to osmotic stress [43]. This osmotic stress causes a change in the intracellular pH and ionic strength, which inactivates enzymes, denatures other proteins, and subsequently interferes with metabolic processes. An increase in the freezing rate can increase the survival of microorganisms by reducing the period over which they are exposed to osmotic stress. In addition, depending on the chemistry and concentration of solutes in unfrozen water, an increase in the freezing rate can cause the solutes to freeze with the water (i.e., freeze as a solution). This reduces the degree of osmotic stress microorganisms are exposed to from the remaining unfrozen water fraction [33, 43]. In the current study, molecular quantification of total bacteria revealed some differences between samples thawed after three days and some of the other time points. Whilst variations between measurements of ~-0.20 log_10_ bacteria/g are of debatable biological significance [14], observed statistical differences could be associated with stochastically different microbial abundance and concentration of solutes in water proteins and carbohydrates amongst the frozen aliquots.

These results are in agreement with previous studies [33, 40–43] that have shown that at -10°C, the ice fraction in meat samples makes up 83%, at -20°C, it reaches 88%, and at -40°C though it is considered entirely frozen, yet around 10% of the water remains unfrozen and is usually associated with the structural proteins [33, 43, 44]. *Campylobacter* is especially sensitive to freezing, with some variation in freezing tolerance between strains of *C*. *jejuni* [40, 41]. It has often been suggested that under environmental stress and unfavourable growth conditions, *C*. *jejuni* enter a viable but non-culturable state [45–48]. Although viability tests were not conducted in the current study, the reduction in concentration via CFU enumeration at day 3 could be likely due to the bacteria entering into the state of dormancy with a relatively reduced growth rate on CCDA while retaining viability [45, 49, 50]. The reduction and the consistent presence of *C*. *jejuni* revealed by both culture and qPCR methods further suggest that freezing samples prior to analysis at -80°C for up to 62 days do not kill *C*. *jejuni*. It can be further speculated that under a favourable environment could increase the recovery rate and, therefore, virulence, giving rise to an important reservoir of infection and public health risk.

The ranking order at day 0 for CFU enumeration was somewhat opposite from the remaining time points, indicating that the samples analysed did not show a constant ranking and therefore the rate at which their relative concentration decreased was not constant throughout them. However, the ranking order for measurements from day 3 onwards was strong-to-very-strong positively correlated, likely indicating that samples had relatively same CFU ratio from day 3 to day 62.

On the other hand, ρ values associated with qPCR pointed towards good replicability of the technique, which was able to estimate a somewhat similar concentration ratio through the samples analysed. Moreover, CFU ranking at D0 was moderately correlated (ρ = 0.46) to D0 ranking for qPCR measures of *C*. *jejuni*, whilst being weakly correlated to ranking of D3-D62 qPCR-*C*. *jejuni*, indicating that albeit retrieving similar concentrations at day 0, the two techniques attributed different relative abundances through the sample population at each time point. Likely, these calculated differences were due to a combination of stochastic variations throughout the samples analysed and technical sensitivity. Whether similar influence of natural stochasticity would mask expected ranking derived from pre-established variation of *in-vivo C*. *jejuni* concentrations remains to be elucidated.

In terms of a sudden decrease in *Campylobacter* concentration upon freezing, our results were in line with researchers who reported that the frozen storage of chicken wings at −20°C and −30°C for 3 days reduced the *C*. *jejuni counts* by 1.3 and 1.8 log_10_ CFU/g, respectively [51]. Similarly, 1 log_10_ CFU/g reduction in *C*. *jejuni* counts was reported in chicken skin, skinned, deboned thigh, and minced meat preparations after 1 day of storage at -22°C, after which (up to 18 d), only a slight decrease was achieved [35]. Studies, both in pure broth cultures [52] and on naturally contaminated broiler carcasses [53], which were frozen and thawed, showed a reduction in the levels of *C*. *jejuni* or *C*. *coli* and this reduction in counts was associated with the fragility of the organisms relative to the freezing process. Our study results are also in line with another study [11] where while using culture-based detection method, it was found that the level of *Campylobacter* was reduced by approximately 1 log immediately after freezing at -20°C and remained relatively constant during the 31 to 220 days of frozen storage whereas the levels remained constant during 7 days of cold storage (3°C).

In general, our results show that whilst culture-based methods demonstrated high reliability at day 0, as demonstrated by the same levels being retrieved through molecular biology means, care should be taken when estimating bacterial concentration after drops of temperature below 0°C. Molecular biology techniques could provide a rapid and reliable alternative, upon isolation of genetic material, able to detect comparable concentration of viable cells to culture-based techniques in fresh samples and constant concentration of DNA associated with *Campylobacter* cells on frozen samples up to 62 days.

## Conclusions

The present study shows that whilst it is always preferable to analyse samples as soon as possible after sample collection, qPCR reveals to be more reliable than culture-based methods when analysing samples stored at -80°C for up to 62 days as the latter is not sensitive to the initial drop in viable counts. Our results contribute to delineating a standard protocol for *C*. *jejuni* quantification in both fresh and frozen samples, favouring molecular techniques, especially for the latter, whilst being aware that viability information is only retrievable through associated culture-based methods. The study also highlights the fact that a reduction in *C*. *jejuni* quantification associated with samples being frozen and thawed prior to analysis must be factored in when reporting to avoid counterfeited results.

## Supporting information

S1 Data (XLSX)

## References

[1] The European Union One Health 2019 Zoonoses Report. EFSA Journal. 2021;19. doi: 10.2903/J.EFSA.2021.6406 PMC7913300 33680134

[2] Facciolà A, Riso R, Avventuroso E, Visalli G, Delia SA, Laganà P. Campylobacter: from microbiology to prevention. Journal of Preventive Medicine and Hygiene. 2017;58. 28900347 PMC5584092

[3] Carron M, Chang YM, Momanyi K, Akoko J, Kiiru J, Bettridge J, et al. Campylobacter, a zoonotic pathogen of global importance: Prevalence and risk factors in the fast-evolving chicken meat system of Nairobi, Kenya. PLOS Neglected Tropical Diseases. 2018;12: e0006658. doi: 10.1371/journal.pntd.0006658 30102697 PMC6122836

[4] Keener KM, Bashor MP, Curtis PA, Sheldon BW, Kathariou S. Comprehensive Review of Campylobacter and Poultry Processing. Comprehensive Reviews in Food Science and Food Safety. 2004;3: 105–116. doi: 10.1111/j.1541-4337.2004.tb00060.x 33430546

[5] Kaakoush NO, Miller WG, de Reuse H, Mendz GL. Oxygen requirement and tolerance of Campylobacter jejuni. Research in Microbiology. 2007;158: 644–650. doi: 10.1016/j.resmic.2007.07.009 17890061

[6] Hickey TE, Majam G, Guerry P. Intracellular survival of Campylobacter jejuni in human monocytic cells and induction of apoptotic death by cytholethal distending toxin. Infection and Immunity. 2005;73: 5194–5197. doi: 10.1128/IAI.73.8.5194-5197.2005 16041038 PMC1201269

[7] Park SF. The physiology of Campylobacter species and its relevance to their role as foodborne pathogens. International Journal of Food Microbiology. 2002;74: 177–188. doi: 10.1016/s0168-1605(01)00678-x 11981968

[8] Lee A, Snith SC, Coloe PJ. Survival and Growth of Campylobacter jejuni after Artificial Inoculation onto Chicken Skin as a Function of Temperature and Packaging Conditions. Journal of Food Protection. 1998;61: 1609–1614. doi: 10.4315/0362-028x-61.12.1609 9874337

[9] Chan KF, le Tran H, Kanenaka RY, Kathariou S. Survival of Clinical and Poultry-Derived Isolates of Campylobacter jejuni at a Low Temperature (4°C). Applied and Environmental Microbiology. 2001;67: 4186–4191. doi: 10.1128/AEM.67.9.4186–4191.2001 11526022 PMC93146

[10] Lázaro B, Cárcamo J, Audícana A, Perales I, Fernández-Astorga A. Viability and DNA maintenance in nonculturable spiral Campylobacter jejuni cells after long-term exposure to low temperatures. Applied and Environmental Microbiology. 1999;65: 4677–4681. doi: 10.1128/AEM.65.10.4677-4681.1999 10508106 PMC91624

[11] Georgsson F, Áorkelsson E, Geirsdóttir M, Reiersen J, Stern NJ. The influence of freezing and duration of storage on Campylobacter and indicator bacteria in broiler carcasses. Food Microbiology. 2006;23: 677–683. doi: 10.1016/j.fm.2005.10.003 16943068

[12] Bullman S O’Leary J, Corcoran D, Sleator RD, Lucey B. Molecular-based detection of non-culturable and emerging campylobacteria in patients presenting with gastroenteritis. Epidemiol Infect. 2012;140: 684–688. doi: 10.1017/S0950268811000859 21676357

[13] Debretsion A, Habtemariam T, Wilson S, Nganwa D, Yehualaeshet T. Real-time PCR assay for rapid detection and quantification of Campylobacter jejuni on chicken rinses from poultry processing plant. Molecular and Cellular Probes. 2007;21: 177–181. doi: 10.1016/j.mcp.2006.10.006 17223308

[14] Mayr AM, Lick S, Bauer J, Thärigen D, Busch U, Huber I. Rapid Detection and Differentiation of Campylobacter jejuni, Campylobacter coli, and Campylobacter lari in Food, Using Multiplex Real-Time PCR. Journal of Food Protection. 2010;73: 241–250. doi: 10.4315/0362-028x-73.2.241 20132668

[15] Khattak F, Pedersen NR, Matthiesen R, Houdijk JGM. Lacto-fermented rapeseed meal additive: a nutritional intervention to reduce Campylobacter jejuni colonisation and improve performance in broilers. British Poultry Abstracts. 2021;17: 7–8.

[16] Khattak F, Paschalis V, Green M, Houdijk GM, Soultanas P, Mahdavi J. TYPLEX® Chelate, a novel feed additive, inhibits Campylobacter jejuni biofilm formation and cecal colonization in broiler chickens. Poultry Science. 2018;97: 1391–1399. doi: 10.3382/ps/pex413 29462463 PMC5914411

[17] Hou Y, Zhang H, Miranda L, Lin S. Serious Overestimation in Quantitative PCR by Circular (Supercoiled) Plasmid Standard: Microalgal pcna as the Model Gene. PlosOne. 2010;5: e9545. doi: 10.1371/journal.pone.0009545 20221433 PMC2832698

[18] de Boer P, Rahaoui H, Leer RJ, Montijn RC, van der Vossen JMBM. Real-time PCR detection of Campylobacter spp.: A comparison to classic culturing and enrichment. Food Microbiology. 2015;51: 96–100. doi: 10.1016/j.fm.2015.05.006 26187833

[19] Muyzer G, De Waal EC, Uitterlinden AG. Profiling of Complex Microbial Populations by Denaturing Gradient Gel Electrophoresis Analysis of Polymerase Chain Reaction-Amplified Genes Coding for 16S rRNA. Applied and Enviromental Microbiology. 1993;59: 695–700. doi: 10.1128/aem.59.3.695-700.1993 7683183 PMC202176

[20] Stoddard SF, Smith BJ, Hein R, Roller BRK, Schmidt TM. rrnDB: improved tools for interpreting rRNA gene abundance in bacteria and archaea and a new foundation for future development. Nucleic Acids Research. 2015;43: 593–598. doi: 10.1093/nar/gku1201 25414355 PMC4383981

[21] Singh KM, Pandya PR, Tripathi AK, Patel GR, Parnerkar S, Kothari RK, et al. Study of rumen metagenome community using qPCR under different diets. Meta Gene. 2014;2: 191–199. doi: 10.1016/j.mgene.2014.01.001 25606402 PMC4287863

[22] R Core Team. R: A Language and Environment for Statistical Computing. Vienna, Austria; 2022. Available: https://www.R-project.org/

[23] Artusi R, Verderio P, Marubini E. Bravais-Pearson and Spearman correlation coefficients: Meaning, test of hypothesis and confidence interval. International Journal of Biological Markers. 2002;17: 148–151. doi: 10.5301/jbm.2008.2127 12113584

[24] Zar JH. Significance testing of the spearman rank correlation coefficient. J Am Stat Assoc. 1972;67: 578–580. doi: 10.1080/01621459.1972.10481251

[25] Hazeleger WC, Beumer RR. Campylobacter: Detection by Latex Agglutination Techniques. Second Edi. Encyclopedia of Food Microbiology: Second Edition. Elsevier; 2014. doi: 10.1016/B978-0-12-384730-0.00054–9

[26] Food Standards Agency. A UK wide microbiological survey of Campylobacter contamination in fresh whole chilled chickens at retail sale (Year 3/ 4). 2016. Available: https://webarchive.nationalarchives.gov.uk/ukgwa/20180411191728/https://www.food.gov.uk/sites/default/files/retail_survey_protocol_year3.pdf

[27] Jorgensen F, Charlett A, Swift C, Corcionivoschi N, Elviss NC. Year 4 Report A microbiological survey of Campylobacter contamination in fresh whole UK-produced chilled chickens at retail sale. 2019. Available: https://www.food.gov.uk/sites/default/files/media/document/campylobacter-contamination-uk-chickens-year-4-report.pdf

[28] Jorgensen F, Charlett A, Swift C, Painset A, Corcionivoschi N. A survey of the levels of Campylobacter spp. contamination and prevalence of selected antimicrobial resistance determinants in fresh whole UK-produced chilled chickens at retail sale (non-major retailers) FSA Project FS102121 Year 5 (2018/19) Report. 2021 [cited 11 Apr 2022]. doi: 10.46756/sci.fsa.xls618

[29] Endtz HP, Ruijs GJHM, Zwinderman AH, van der Reijden T, Biever M, Mouton RP. Comparison of Six Media, Including a Semisolid Agar, for the Isolation of Various Campylobacter Species from Stool Specimens. Journal of Clinical Microbiology. 1991;29: 1007–1010. doi: 10.1128/jcm.29.5.1007-1010.1991, Available: http://jcm.asm.org/ 2056033 PMC269924

[30] Piersimoni C, Bornigia S, Curzi L, de Sio G. Comparison of two selective media and a membrane filter technique for isolation of Campylobacter from diarrhoeal stools. European Journal of Microbiology and Infectious Diseases. 1995;14: 539–542. Available: doi: 10.1007/BF02113436 7588831

[31] Ohkoshi Y, Sato T, Murabayashi H, Sakai K, Takakuwa Y, Fukushima Y, et al. Campylobacter upsaliensis isolated from a giant hepatic cyst. Journal of Infection and Chemotherapy. 2020;26: 752–755. doi: 10.1016/j.jiac.2020.02.015 32199791

[32] Maher M, Finnegan C, Collins E, Ward B, Carroll C, Cormican M. Evaluation of Culture Methods and a DNA Probe-Based PCR Assay for Detection of Campylobacter Species in Clinical Specimens of Feces. Journal of Clinical Microbiology. 2003;41: 2980–2986. doi: 10.1128/JCM.41.7.2980-2986.2003 12843030 PMC165355

[33] Gill CO. Microbial control with cold temperatures. Control of Foodborne Microorganisms. 2001; 55–74. doi: 10.1201/B16945-3

[34] International Commission on Microbiological Specifications for Foods. Micro-organisms in foods 6: microbal ecology of food commodities. 2005; 763.

[35] Sampers I, Habib I, de Zutter L, Dumoulin A, Uyttendaele M. Survival of Campylobacter spp. in poultry meat preparations subjected to freezing, refrigeration, minor salt concentration, and heat treatment. International Journal of Food Microbiology. 2010;137: 147–153. doi: 10.1016/j.ijfoodmicro.2009.11.013 20006911

[36] Keer JT, Birch L. Molecular methods for the assessment of bacterial viability. Journal of Microbiological Methods. Elsevier; 2003. pp. 175–183. doi: 10.1016/S0167-7012(03)00025-3 12654489

[37] Oh E, Andrews KJ, McMullen LM, Jeon B. Tolerance to stress conditions associated with food safety in Campylobacter jejuni strains isolated from retail raw chicken. Scientific Reports 2019 9:1. 2019;9: 1–9. doi: 10.1038/s41598-019-48373-0 31417115 PMC6695378

[38] Bang DD, Madsen M. Investigation of the Campylobacter jejuni Cold-Shock Response by Global Transcript Profiling. Genome Letters. 2003;2: 73–82. doi: 10.1166/GL.2003.000

[39] Sutton S. The limitations of CFU: Compliance to CGMP requires Good Science. Journal of GXP Compliance. 2012;16: 74–80.

[40] Micro-Organisms in Foods 6. Micro-Organisms in Foods 6. 2005. doi: 10.1007/0-387-28801-5

[41] Archer DL. Freezing: an underutilized food safety technology? International Journal of Food Microbiology. 2004;90: 127–138. doi: 10.1016/s0168-1605(03)00215-0 14698095

[42] Ayres JC, Mundt JO, Sandine WE. Microbiology of foods. Microbiology of foods. 1980.

[43] Farkas J. Physical methods of food preservation. In: Doyle MP, Beuchat L R, Montville TJ, editors. Food microbiology: fundamentals and frontiers. Washington D. C.: ASM Press; 1997. pp. 497–519.

[44] Bhaduri S, Cottrell B. Survival of cold-stressed Campylobacter jejuni on ground chicken and chicken skin during frozen storage. Applied and Environmental Microbiology. 2004;70: 7103–7109. doi: 10.1128/AEM.70.12.7103-7109.2004 15574906 PMC535211

[45] Moore JE. Bacterial dormancy in Campylobacter: abstract theory or cause for concern? International Journal of Food Science & Technology. 2001;36: 593–600. doi: 10.1046/J.1365-2621.2001.00508.X

[46] Murphy C, Carroll C, Jordan KN. Environmental survival mechanisms of the foodborne pathogen Campylobacter jejuni. Journal of Applied Microbiology. 2006;100: 623–632. doi: 10.1111/j.1365-2672.2006.02903.x 16553716

[47] Pearson AD, Greenwood M, Healing TD, Rollins D, Shahamat M, Donaldson J, et al. Colonization of broiler chickens by waterborne Campylobacter jejuni. Applied and Environmental Microbiology. 1993;59: 987–996. doi: 10.1128/aem.59.4.987-996.1993 8476300 PMC202227

[48] Rollins DM, Colwell RR. Viable but nonculturable stage of Campylobacter jejuni and its role in survival in the natural aquatic environment. Applied and Environmental Microbiology. 1986;52: 531–538. doi: 10.1128/aem.52.3.531-538.1986 3767358 PMC203568

[49] Barer MR, Harwood CR. Bacterial Viability and Culturability. Advances in Microbial Physiology. 1999;41: 93–137. doi: 10.1016/s0065-2911(08)60166-6 10500845

[50] OJ D. The Viable but Nonculturable State in Bacteria. Journal of Microbiology. 2005;43: 93–100. 15765062

[51] Zhao T, Ezeike GOI, Doyle MP, Hung YC, Howell RS. Reduction of Campylobacter jejuni on Poultry by Low-Temperature Treatment. Journal of Food Protection. 2003;66: 652–655. doi: 10.4315/0362-028x-66.4.652 12696690

[52] Humphrey TJ, Cruickshank JG. Antibiotic and deoxycholate resistance in Campylobacter jejuni following freezing or heating. Journal of Applied Bacteriology. 1985;59: 65–71. doi: 10.1111/j.1365-2672.1985.tb01777.x 3928571

[53] Stern NJ, Rothenberg PJ, Stone JM. Enumeration and Reduction of Campylobacter jejuni in Poultry and Red Meats. Journal of Food Protection. 1985;48: 606–610. doi: 10.4315/0362-028X-48.7.606 30943628

